# When Less Is Best: Female Brown-Headed Cowbirds Prefer Less Intense Male Displays

**DOI:** 10.1371/journal.pone.0036130

**Published:** 2012-05-02

**Authors:** Adrian L. O'Loghlen, Stephen I. Rothstein

**Affiliations:** Department of Ecology, Evolution and Marine Biology, University of California Santa Barbara, Santa Barbara, California, United States of America; University of Manitoba, Canada

## Abstract

Sexual selection theory predicts that females should prefer males with the most intense courtship displays. However, wing-spread song displays that male brown-headed cowbirds (*Molothrus ater*) direct at females are generally less intense than versions of this display that are directed at other males. Because male-directed displays are used in aggressive signaling, we hypothesized that females should prefer lower intensity performances of this display. To test this hypothesis, we played audiovisual recordings showing the same males performing both high intensity male-directed and low intensity female-directed displays to females (N = 8) and recorded the females' copulation solicitation display (CSD) responses. All eight females responded strongly to both categories of playbacks but were more sexually stimulated by the low intensity female-directed displays. Because each pair of high and low intensity playback videos had the exact same audio track, the divergent responses of females must have been based on differences in the visual content of the displays shown in the videos. Preferences female cowbirds show in acoustic CSD studies are correlated with mate choice in field and captivity studies and this is also likely to be true for preferences elucidated by playback of audiovisual displays. Female preferences for low intensity female-directed displays may explain why male cowbirds rarely use high intensity displays when signaling to females. Repetitive high intensity displays may demonstrate a male's current condition and explain why these displays are used in male-male interactions which can escalate into physical fights in which males in poorer condition could be injured or killed. This is the first study in songbirds to use audiovisual playbacks to assess how female sexual behavior varies in response to variation in a male visual display.

## Introduction

Visual displays involving motion are likely to be physiologically costly especially when performed repeatedly, as is often the case with the audiovisual (AV) courtship displays of many avian and other species. Sexual selection theory predicts that these types of energetically demanding displays will be used by females in mate choice because display vigor or skill, or both, could be an honest indicator of a male's current physical condition, which is likely to be related to his genetic quality [1], [2], [3] and the quality of his parental care if such care is provided. Thus, in theory, a female that chooses a mate based on his superior display performance should have higher fitness than a less choosy female because her offspring will inherit higher quality genes from their father. In these circumstances, males will be selected to produce display performances that optimize the trade-off between their mating success and the costs of the display (such as its effects on survival), and higher-quality males will produce the most physiologically demanding display performances. In turn, females should choose a high-quality mate on the basis of how he performs his courtship displays.

Consistent with this hypothesis, males in some species adjust their courtship displays in response to environmental (e.g. light levels, predators) or social factors (e.g. presence of rival males) that affect the costs and benefits of displaying [2]. However, males may also facultatively adjust their display performances in response to females' reactions to these displays. For example, male satin bowerbirds (*Ptilonorhynchus violaceus*) reduce the intensity of their courtship displays in response to the ‘startle’ reactions of females [4]. These male displays in satin bowerbirds are also used as aggressive signals in interactions with rival males and it has been proposed that more intense displays appear threatening to females and inappropriate for courtship. So, male satin bowerbirds may not always display at the maximum intensity that they are capable of so as to avoid startling females and disrupting courtship.

We have suggested that male brown-headed cowbirds (*Molothrus ater*) face a similar dilemma to satin bowerbirds but have evolved a different strategy to resolve it [5]. Perched songs are one of two categories of structurally distinct cowbird songs [6], [7] that are used in intra- and intersexual interactions and variation in perched songs influences female mate choice and thus male mating success [7], [8], [9], [10], [11]. Perched songs are commonly used when birds are in close proximity (<0.5 m) and male cowbirds have repertoires of two to eight different perched song types that share key acoustic features and are frequently given in long bouts directed at other males or females [12], [13], [14].

Perched songs are sometimes broadcast with no other cowbird nearby, but when directed at a nearby cowbird of either gender, they are typically accompanied by a wing-spread display (see Videos S1, S2 in Supporting Information section) that may be very elaborate and end with the male in a head down bowed position [5], [15], [16]. Although earlier studies noted variation in the performance of this display [17], [18], the extent of this variation was only recently quantified using slow motion playback (Videos S3, S4) of video recordings [5]. In this latter study, quantification of display variation was based mainly on scoring the intensity with which display components were performed, e.g., the depth of the bowing motion, rather than presence or absence of display components. We reported that the intensity with which displays are performed varies based on social context and contrary to theoretical prediction, displays directed at females are produced at a lower intensity and are more variable that those directed at other males [5]. Furthermore, display intensity is based largely on social context and less or not at all on responses to the receivers' reactions, unlike the case with satin bowerbirds. We also reported that there was no overlap between average scores for displays directed at other males versus at females and suggested that male- and female-directed displays may be distinct signals. We present evidence in the current study that contradicts this latter suggestion and supports the notion that these displays are one signal that varies in intensity.

Male cowbirds establish dominance in part by counter-singing with rival males in protracted bouts of directed perched songs [10], [16] which on rare occasions may escalate into physical conflict [19]. High-intensity wing-spread displays are clearly an integral part of these aggressive signaling interactions and variation in display intensity based on social context may have evolved because male cowbirds have been selected to avoid these aggressive signals when courting females. Accordingly, and as described in Experiment 1 below, we tested the hypothesis that female cowbirds should be more sexually stimulated by the low intensity performances of the wing-spread song display typically directed at females than by the higher intensity versions usually directed at males.

Numerous previous studies have demonstrated that audio playback of perched songs is an extremely reliable stimulus for eliciting CSDs from female cowbirds [6], [20], [21], [22], [23], [24], and results from a recent study demonstrated that the addition of the visual component of the wing-spread display to the acoustic component enhances female sexual responses [25]. The evidence that the audio component of wing-spread song displays alone can elicit CSDs from females, prompts the question of whether the visual component alone also elicit CSDs? This question is relevant to the present study because if the visual part of the male display is not a stand-alone signal, its function may be to modulate the information provided by the song. If this is the case, the observed variation in the intensity of the visual component of the display may determine whether the message is one of aggression or of courtship. Based on the evidence that cowbirds may produce perched songs with little or no visual display when they sing alone [14], [15], but are not known to perform wing-spread displays without an accompanying perched song, we predicted that females would be more responsive to playback of AV recordings of displays with both sound and visual components versus identical playbacks without sound and tested this prediction as described in Experiment 2 below.

## Materials and Methods

We recently developed experimental CSD procedures that demonstrate that female cowbirds extract meaningful visual information from AV recordings played on a LCD monitor [25] and we used these procedures in the current study. In Experiment 1, we recorded and measured the duration of female CSD responses to playback of AV recordings showing the same males performing both high intensity male-directed and low intensity female-directed wing-spread displays. In Experiment 2, we presented the same females with different AV recordings of low intensity female-directed displays played with and without an accompanying perched song audio track to determine whether the visual information alone in a wing-spread display elicits female CSDs that are as strong as those elicited by AV playbacks.

### Female subjects

We used eight female brown-headed cowbirds that had been trapped as juveniles near Mammoth Lakes, Mono Co., California, in 2005, as subjects in this experiment. During their first year in captivity these females were housed in groups with other females but without males. For the rest of the time prior to the current experiment in 2010, the females were housed with males and other females in outdoor flight cages (approx. 1.2×2.7×6.0 m) except for brief periods each year in 2006 to 2008 inclusive, when they participated in other CSD experiments during which they spent 10–15 days housed in individual isolation chambers. Females were tested in two cohorts of four birds from 3^rd^ to 30^th^ May 2010. Each female had a Silastic tubing implant (outer diameter 1.96 mm) packed with 10 mm of estradiol (Sigma Chemical Co., St. Louis, MO) and sealed with Silastic adhesive, inserted subcutaneously in her chest to increase her sensitivity to playback stimuli [6], [20], [21], [22], [25]. Levels of circulating estradiol in implanted female songbirds are generally within natural physiological bounds and comparable to normal peaks that occur in passerine birds during the period when they are laying eggs [26]. There is no evidence that the increased levels of estradiol from implant procedures affect discrimination in CSD tests of female song preferences [27]. After this procedure, females were transferred to individual cages (46×27×27 cm), which were placed in separate acoustic-isolation chambers (inner dimensions, 61×33×38 cm) as described elsewhere in detail [25]. On the 11^th^ day after receiving her implant, a female was temporarily transferred in her cage to a large sound-attenuating chamber (inner dimensions, 52×57×56 cm), equipped for AV recording and playback where she was presented with a single playback and her response was recorded on digital video. She was then returned to her chamber and another female was run through the test procedure. Females were tested once every hour with a different AV recording as described in detail below.

We ran the females through mock tests 2 or 3 days before their experimental tests started to accustom them to the test procedures. During these mock tests, females were rotated in and out of the AV chamber on the same hourly schedule as on a test day, but instead of being presented with playbacks of males performing song displays, they were shown videos of a caged female cowbird moving about and feeding. Females were given three or four mock trials per day over 2 days.

We used an outdoor photocell switch to maintain the natural photoperiod of the local area in the isolation chambers and females had access *ad libitum* to food (Mazuri Small Birds Maintenance Kibble) and water.

### Recording female responses

The AV chamber in which the females were tested was fitted with a 43 cm Dell E176FP LCD monitor, a Logitech QuickCam 9000 USB webcam, two stereo computer speakers (Cyber Acoustics CA-2014), a 30.5 cm-long 24 LED light fixture, and an external air pump. The webcam was attached to a Dell D420 laptop computer running Windows XP and PBcam software (J. Burt). The computer recorded the females' responses to the playbacks and saved the recordings as avi files (video, 640×480 pixels, 20 frames per second [fps], 24 bit, Huffyuv lossless compression; audio, 44.1 kHz, 16 bit, Pulse Code Modulation [PCM]). AV test recordings were presented to the females on the LCD monitor and stereo speakers, which were attached to a Dell Inspiron 9300 laptop running Windows XP and VirtualDub (A. Lee).

### Playback recordings and procedures

We used wing-spread displays recorded from seven of nine male subjects in a previous study [5] as playback stimuli in the current study. These seven males were chosen based on the quality of their recordings. Details of these males and procedures used to obtain the AV recordings (video, 640×480 pixels, 24 bit color, 30 fps, Huffyuv lossless compression; audio, 44.1 kHz, 16 bit, PCM) of male- and female-directed wing-spread displays are described in O'Loghlen and Rothstein [5]. Male cowbirds in these recordings (see links to videos at Supporting Information section) are shown directing wing-spread displays directly at the camera and these displays are seen from the perspective of a conspecific receiver when played back although the receiver is not visible in these recordings.

**Figure 1:**
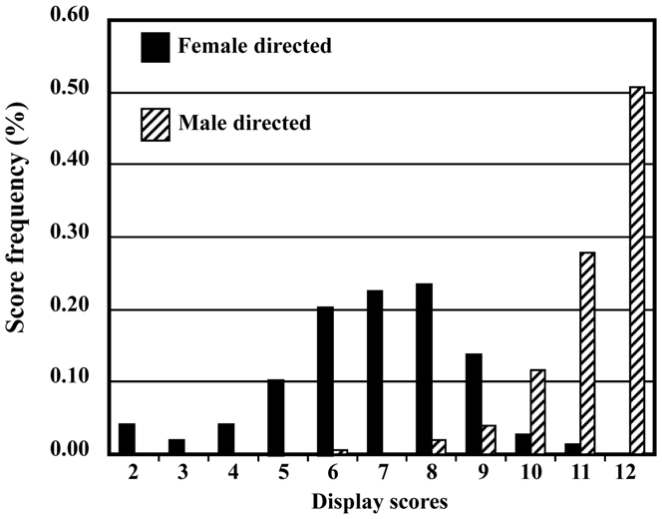
Histogram of the frequency distributions of scores for all female- (n = 148) and male-directed (n = 151) wing-spread song displays recorded from the 7 male brown-headed cowbirds that featured in the playback video stimuli presented to the female subjects. Recordings used as stimuli were selected from these recordings as described in the text. Note the overlap in the distribution of scores for male- and female-directed displays.

We quantified the intensity of male- and female-directed displays from the video recordings using a scoring scheme based on five independent visual components of the display [5]. Displays start with the male ‘puffing up’ his head and chest feathers, spreading and pumping his wings and finally bowing to the receiver as he retracts his wings [5], [15], [17]. The intended recipient of a wing-spread display is always obvious because males face the receiver, often turning abruptly to do so, as they begin to puff their feathers. Male-directed displays in a previous study [5] were significantly longer, more intense (mean score range 10–12, out of a maximum possible 12, n = 9 males) and involved more extreme motions (wider wing spreads, deeper bows) than female-directed displays (mean score range 4–8). We used display scores from recordings made for that study to calculate a median score for each male's male- and female-directed displays respectively. In that study average scores were based on the last six displays a male directed at a conspecific stimulus bird during a recording session whereas the larger samples shown here in Table 1 and Fig. 1 include all recordings in which it was possible to score a male's display fully. Each male was given the opportunity to direct wing-spread displays at 3 different male and 3 different female stimulus birds (each presented alone), although not all male subjects sang to all the stimulus birds [5]. For playbacks, we chose a male- and a female-directed display recording from each male that had scores that matched his separate median scores for each gender. So in total, there were 14 playback display recordings, two from each of the seven males, with one recording consisting of a male-directed display and the other of a female-directed display.

**Table 1:** Median scores of female- and male-directed wing-spread displays for each of the seven males appearing in video recordings used as playbacks to the female cowbirds.

Male in video recording	Male wing-spread song displays					
	Female-directed			Male-directed		
	Median score	Range of scores	# of displays	Median score	Range of scores	# of displays
SRx4	4	2–6	24	10	6–12	22
SC#18	6	5–7	19	12	8–12	16
SC#17	7	5–9	17	12	10–12	22
SC# 16	7	5–11	24	12	11–12	22
Vx4	8	6–10	25	12	9–12	25
DBx4	8	3–11	17	11	10–12	20
RBx4	8	6–9	22	11	11–12	24
Mean	7.0	2–11	21.1	11.4	6–12	20.1

The numbers of video recordings of displays and range of display scores on which the median scores for each male were based are also shown.

Using VirtualDub, we edited the two videos in a pair so that both were approximately the same length (means ±S.E. duration; low intensity 3.35±0.27 s, high intensity, 3.37±0.27 s). Because the original acoustic recordings of the perched songs on the videos were of poor quality, we stripped the audio tracks from all the videos and added a new higher quality sound track (44.1 kHz, 16 bit, PCM) to each one [25]. The same audio file of a single perched song was added to each of the two videos in a pair and a different perched song type was used for each of the seven video pairs. The duration of audio and video tracks were virtually identical so that when they were merged together, the songs played at the same time as the males displayed but display motions and songs were not as accurately synchronized as occurs in natural singing [15]. Three of the perched songs used were recorded from two of the males that appeared in the videos but these songs were not matched with the males that produced them. We added ‘fade in’ and ‘fade out’ visual effects to all playback videos so that they started and finished with a darkened screen. In summary, there were seven pairs of AV recordings with the same male in each pair performing either a high intensity male-directed or low intensity female-directed wing-spread display with both video recordings having the exact same audio track with a different song for each video pair.

For Experiment 2 (video stimuli with versus without audio), we chose 6 AV recordings (video, 800×600 pixels, 24 bit color, 20 fps, uncompressed; audio, 44.1 kHz, 16 bit, PCM) of female-directed displays obtained from five different males. Five of these videos had been used as playbacks in a previous study [25] and the remaining video was a second recording of one of these males which was not used in that study but was recorded under the same conditions and edited in the exact same manner as the other videos. One of the males featured in the videos was also in the recordings used in Experiment 1 and the other four were different males.

### Playback procedures

We used VirtualDub software to present each of the eight females with six of the seven video pairs of high intensity male-directed and low intensity female-directed display recordings described above (Experiment 1). A single AV recording was played to each female once an hour from 08:00 through 13:00 PST on each of the first two test days (six per day) for a total of 12 playbacks. Although male cowbirds are often observed directing a long series of wing-spread displays at females during courtship, these are not the circumstances under which CSDs and copulations occur in the wild. Copulations in nature typically involve a male that was spatially separated from a female (but had previously courted the female extensively [10], [14]), flying to the perched female and directing up to at most 3 perched songs at her in the seconds immediately prior to or during copulation [8], [9]. Thus, our use of a single song display to elicit a CSD is within the range of male behavior associated with copulations in nature. The two AV playback videos showing the same male performing a low intensity female-directed display in one and a high intensity male-directed display in the other were alternated for the three pairs of videos shown each day and the order (high versus low intensity) was varied for each female on the two test days and by individual female to avoid any systematic biases. Because our experimental design which was based on our extensive past experience with acoustic CSD studies, was restrained by the number of times we could test a female before she was likely to be affected by habituation to the test procedures and by the fact that we confined tests to the mornings when the females are most responsive, not all 8 females viewed all 7 video pairs. Thus, 3 of the 7 videos pairs were viewed by all 8 females, one pair by 7, one pair by 6, and the remaining two video pairs by 5 females.

We tested female responses to videos of low intensity female-directed displays with and without audio (Experiment 2) on test Day 3 exclusively. Females were presented with six videos following the procedures described above, three of which were AV display recordings and three the same video recordings without audio (‘no audio’ setting in VirtualDub). The ‘with’ and ‘without audio’ pairs were alternated in our playbacks and the order was varied to avoid any systematic bias. The first cohort of four of the females was presented with videos showing three different males displaying and the second cohort saw recordings from two other males plus a display recording from a male shown to the first cohort. As explained above, the latter was a different recording of this male than that used with the first cohort.

In all playback presentations, video size was set at 150% of normal in VirtualDub and the image did not fill the entire monitor screen. This setting was chosen so that males in the videos were approximately life size.

### Statistical analyses

As in previous studies of copulation solicitation displays (CSDs), we used the average duration of displays elicited from females to each stimulus category as our response variable [20], [21], [22], [25], [28]. Using VirtualDub software, AOL viewed all the recordings of female responses on a Dell Inspiron 9300 laptop. CSD responses were measured in frame-by-frame playback and durations were determined mainly on the basis of tail position, as in O'Loghlen and Rothstein [6]. CSD displays in cowbirds [29], [30] and other songbirds [31] are clear and unambiguous, and the intensity of a display is correlated with its duration. Independent assessment of CSD display durations is redundant, as separate repeated measurements have confirmed the reliability of the procedures we describe for determining duration [7], [21], [31]. Because the original measurement of female response durations was not conducted ‘blindly’ with respect to display category (high versus low intensity), duration measurements were repeated on 16 video recordings (eight high and low intensity displays respectively with four videos from each of four females) by AOL without knowledge of the display intensity in the playback video. These latter durations were very strongly correlated with the original durations (Spearman rank-order correlation coefficient r_s_ = 0.97, N = 16, P<0.0001). We used a Wilcoxon signed rank test to compare the average duration of each female's response to playbacks of high intensity male-directed displays with her average for low intensity female-directed displays on Days 1 and 2 (Experiment 1) and to compare CSD durations elicited by the video recordings played ‘with’ and without’ perched song audio tracks on test Day 3 (Experiment 2). We calculated effect size estimates [r, 32] for both these analyses using *z* values as described in Fritz et al. [33].

Video playbacks used on Day 3 were recorded at different frame rates and resolution (20 fps, 800×600 pixels) than those used on Day 1 and 2 (30 fps, 640×480 pixels), and to investigate whether these differences affected females' responses, we compared durations of female displays elicited by AV playbacks of low intensity male displays on Day 3 with the equivalent durations obtained on Day 1 and 2, again using a Wilcoxon signed rank test. Results are means ± SE. We used Statistica (StatSoft) software, and probabilities were two-tailed.

### Ethics statement

Birds used in this study were trapped, banded, and maintained in captivity under the appropriate federal and state permits. This study was carried out in strict accordance with the recommendations in the Guidelines to the Use of Wild Birds in Research published by The Ornithological Council. The research protocol used was approved by the Institutional Animal Care and Use Committee of the University of California, Santa Barbara (Protocol # 185).

## Results

### Scores for male displays used as playback stimuli

Scores for female-directed displays ranged from 2 to 11 and for male-directed displays from 6 to 12 (Fig. 1). The maximum possible score was 12 and medians were based on an average of 21.3±1.0 male-directed and 21.6±1.2 female-directed displays per male (N = 7). Median scores for individual male's low and high intensity displays ranged from 4 to 8 and from 10 to 12 respectively, and the two videos recorded from each male that were used as playback stimuli featured displays with scores that matched his low and high intensity median scores (Table 1).

### Female CSD responses to playback of male wing-spread displays

**Figure 2:**
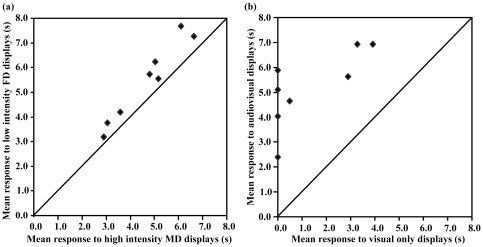
Mean duration of copulation solicitation displays (CSDs) for each female brown-headed cowbird (*n* = 8) elicited in response to playback of video recordings of males performing wing-spread song displays. a) Female CSD responses to recordings of males performing low intensity female-directed (FD) versus high intensity male-directed (MD) displays (Experiment 1, test days 1 and 2), and b) CSD responses to audiovisual recordings of low intensity female-directed displays versus the same recordings played without the audio track of the accompanying perched song (Experiment 2, test day 3). Diagonal lines represent points at which mean response durations are equal.

All eight females responded with copulation solicitation displays to all of the AV stimuli presented to them over the three test days but not to all the video-only playbacks. In Experiment 1, females responded to both high intensity male-directed and low intensity female-directed displays but all were significantly more sexually stimulated by videos showing low intensity female-directed displays (mean CSD duration 5.4±0.6 s, median 5.6, range 3.2–7.7) than high intensity male-directed displays (Fig. 2a, mean 4.7±0.5 s, median 4.9, range 2.9–6.7. Wilcoxon signed ranks test, *z* = 2.52, *t* = 0.0, *p* = 0.01, *r* = 0.89, *n* = 8). According to Cohen's [32] guidelines for interpreting *r*, values >0.5 can be described as large effects. However, other than in the context of demonstrating female sexual preferences among categories of AV playback stimuli, the biological significance of these effect size estimates is unclear because we do not know the precise quantitative relationship between variation in CSD durations in playback experiments and variation in female mate choice and male copulation success in nature.

In Experiment 2 on Day 3, all the females responded to AV playback of low intensity female-directed wing-spread displays, while only four (50%) did so for the video only playbacks of these recordings (Fig. 2b). Females were significantly more stimulated by the AV playbacks with solicitation displays that averaged 5.2±0.5 s (median 5.4, range 2.4–6.9) compared to 1.3±0.6 s for the visual only playbacks (median 0.2, range 0–3.9. Wilcoxon signed ranks, *z* = 2.52, *t* = 0, *p* = 0.01, *r* = 0.89, *n* = 8). Female responses to AV playbacks of low intensity female-directed displays on Day 3 did not differ significantly from those for the equivalent playbacks on Days 1 and 2 (Wilcoxon signed ranks, *t* = 10, *p* = 0.26, *n* = 8,).

## Discussion

Although all the females responded strongly to playback of both high intensity male-directed and low intensity female-directed wing-spread displays in Experiment 1, they were, as predicted, more sexually stimulated by viewing low intensity female-directed versions of this display. Moreover, because each video playback in a test pair had the exact same audio track, differences in responses can only be the result of differences in the visual content of the video pairs, the most obvious of which were the differences in display intensity. These results are consistent with our hypothesis that male cowbirds have been selected to avoid using high intensity wing-spread displays when singing to females because these displays are not optimal courtship signals. As stated previously, sexual preferences shown by female cowbirds in CSD experiments using audio stimuli (flight whistles and perched songs) are correlated with male mating success in lab and field settings [7], [8], [9], [10] and it is reasonable to assume that this association also applies to CSD preferences demonstrated in AV playback studies. Accordingly, males that court females using displays with an appropriately low level of intensity should have greater mating success than males that use high intensity displays during courtship and this may be a reason for the differences in male display intensities based on social context [5].

Females' strong sexual responses to both categories of displays contradicts the suggestion we previously made based on the absence of overlap in scores for intensity between male- and female-directed displays, that each of these two categories of displays represent distinct signals [5] and are more consistent with the idea that these displays are just one signal that varies in intensity. Data presented in Fig. 1 based on a larger sample to that on which we made our original proposal also support the latter conclusion because they demonstrate that there is overlap in the intensity scores of displays directed at males and at females.

In a previous study, we suggested that female preferences for AV playbacks showing ‘displaying’ versus ‘non-displaying’ males may have been influenced by the lack of motion, and specifically beak movement, when the accompanying perched song was played with the ‘non-displaying’ male videos [25]. Both categories of videos (low intensity female-directed and high intensity male-directed displays) in the current study featured males performing all the motions, including beak movements, associated with natural songs and wing-spread displays and variation in the intensity of displays presented to the females was consistent with that they were likely to experience in nature (Fig. 1). In the past, technical limitations have hindered attempts to use AV playbacks in songbird communication studies [34], [35], [36], [37] and this is the first study to demonstrate that variation in a male visual display presented in AV playbacks can influence female sexual behavior in a songbird.

Female responses to the video presentations of wing-spread displays ‘with’ and ‘without audio’ (Experiment 2) clearly demonstrate that playback of AV recordings are significantly more stimulating than playback of the same video recordings without the accompanying perched songs. Audiovisual playback studies in other songbirds using different behavioral response measures (e.g. time spent viewing, zebra finch, *Taeniopygia guttata* [38]), have found similar results showing response enhancement when visual and audio signals are combined [39], [40]. The comparatively low responsiveness shown by female cowbirds to the silent videos may suggest that the visual component of the wing-spread display is not as important to females as the acoustic part and that the visual display is not a ‘stand-alone’ signal, which is also supported by the fact that males never perform wing-spread displays without simultaneously singing. Nevertheless, it is clear from the results of the present and an earlier study [25] that females are more sexually stimulated by playback of perched songs that are accompanied by recordings of displaying males than by the same songs played on their own, i.e., audio only. However, it may not be valid to conclude that the low level of female response to the silent video playbacks is entirely a result of the absence of song. In the silent videos, males are seen performing a display that in nature would always be accompanied by a perched song. In these videos, the males' beaks move as if they were producing song and they perform other motions (e.g. wing-spreads, bows, etc.) that are normally tightly integrated with the acoustic component of these displays [15]. As a result, the silent videos of males displaying represent stimuli that females would never experience under natural conditions and this may have affected their responses adversely.

### Possible limitations of using audiovisual stimuli in playback studies

We do not know exactly what females are seeing when they view video recordings on a computer monitor and it is possible that important information is lost due to technical limitations of the recording and playback procedures [41]. Most birds are tetrachromatic possessing ultraviolet (UV) sensitive cone cells in the eye as well as cells for red, green and blue [42] and the webcams used in the current study are not designed to record UV light, which would not be reproduced by a typical computer LCD screen. This limitation is unlikely to have affected female responses in our study because there is no evidence for enhanced UV reflectance in male brown-headed cowbird plumage [43]. The videos used as playback in Experiment 1, the display intensity experiment, were recorded at 30 frames per s, which is a standard for videos and movies designed for human viewing, but motion in these videos may have appeared ‘jerky’ or blurred to the females because birds can resolve rapid movements better than humans [41], [44]. Nevertheless, whatever the visual limitations of our playback procedures were from the females' perspective, the females did distinguish between the two categories of displays based on the visual content alone, and in addition, showed no differences in responsiveness to AV presentations of low intensity female-directed displays presented at 30 fps on Days 1 and 2, and 20 fps on Day 3.

### Why do male cowbirds use low intensity displays to court females?

It is generally accepted that song in birds has dual intraspecific functions; repelling rival males, and attracting and stimulating females [2], [45]. It is also generally accepted, or assumed, that the acoustic structure of a passerine song variant or type does not change when the song is given in either of these contexts although as far as we know this has not been tested explicitly (but see [46], [47]). As yet, we have been unable to detect any acoustic differences based on social context in the perched songs of cowbirds (unpublished data). However, sexual selection theory predicts that the function and information content of male- versus female-directed sexual signals should differ [48], [49]. This apparent contradiction would be resolved if variation in the visual component of cowbird wing-spread displays modulates the acoustic component by providing information appropriate to the social context. For example, a perched song type accompanied by one category of display or display intensity would provide information relevant to female mate choice while the same perched song type and a more intense display would provide information to a rival male about the sender's current physical condition or aggressive intentions. The current results provided indirect support for this proposal because, as far as we can determine, the only information available to the females concerning the social circumstances under which the videos were recorded was the variation in display intensity, and all of the females reacted to the variation in intensity with behavioral responses appropriate to the social context, i.e., they were more sexually stimulated by the female-directed displays.

We have hypothesized that a reason male cowbirds direct higher intensity displays at males relates to differences in the information males are selected to provide when signaling to other males versus females [5], [25]. Signals that involve motion, especially if performed repetitively and at high intensity, as is the case with male-directed displays in cowbirds, are expected to be energetically demanding [2]. Cooper and Goller [15] provided indirect evidence for display costs in cowbirds by demonstrating that the performance of these AV displays is biomechanically constrained. A male cowbird may use the intensity and persistence of display behavior to assess the current physical condition or fighting ability of a rival and benefit from this information by not escalating aggressive interactions with superior males [2], interactions which on rare occasions may escalate into physical combat [19]. However, current condition may not be a reliable basis for females when it comes to choosing a mate. Cowbirds are brood parasites and males do not provide parental care or territories, and the main benefits of mate choice for female cowbirds are likely to be genetic [50], as in many other songbirds and species [51]. Dynamic signals such as the visual component of wing-spread displays are more susceptible to short-term environmental variation and dishonesty than static signals that develop over an extended period and are likely to be a more reliable indicator of a male's genetic quality [52], [53], [54], [55], [56]. Accordingly, the intensity of wing-spread displays is unlikely to be the most dependable source for information available to female cowbirds about a male's genetic quality and females may use other traits when choosing a mate. If the latter is true, this would explain why female-directed displays are so variable when compared to male-directed displays. In addition, males that use low intensity displays in a courtship context may be more successful in obtaining copulations than males that use higher intensity displays because females may perceive the latter males as a physical threat to them [4], [57].

Besides display intensity there are two other potentially more reliable sources of information about a displaying male's genetic quality that are available to a female. One is his repertoire of perched song types and the other is the color of his underwing covert feathers. Both are age-revealing signals or cues that do not change during the course of a breeding season [12], [13], [58] and could be used by females, either separately or in combination, in their demonstrated preference for choosing adult (2 years or older) versus yearling males as mates [21], [59]. Older males in most species are generally considered to be of higher average genetic quality than younger males simply because they have survived for longer [54], [60], although this assertion has been challenged [61]. Furthermore, field and lab studies indicated that female cowbirds' preference for adult mates is, at least in part, influenced by differences between adult and yearling song repertoires [11], [21], [59], [62]. Adult males have larger repertoires of perched song types than yearlings and the vast majority of the adult song types are shared with other adults resident in a local population. In contrast, the majority of yearlings have smaller repertoires that are mainly composed of ‘unique’ song types not found in the repertories of other local males [6], [13]. The proposition that the acoustic component of a wing-spread display is more important to females than the visual component is supported by results from previous studies showing that females respond more reliably to playback of perched songs alone [6], [21], [22] than they do to silent video recordings of displaying males (current study).

Male adult and yearling cowbirds are also generally distinguished by differences in the color of the covert feathers that line the underside of their wings [58], [63]. Adult males typically have dark blue/black covert feathers that match the rest of their plumage whereas most yearlings retain some or all of the light brown covert feathers from their juvenile plumage phase [57]. These juvenile feathers are obvious during wing-spread displays including female-directed displays in which the wings are only partially opened, but are hidden in other situations. So when a male directs a wing-spread display at a female he is essentially advertising his age and females may require males to perform these displays during courtship for this reason. If the reason males direct wing-spread displays at females is to reveal their age, this would also explain why female-directed displays are performed at lower intensities than male-directed displays. Moreover, plumage differences may be a better cue than song repertoires for females to use when choosing an adult mate because yearling covert feathers are a more reliable indicator of age. Whereas a small proportion of yearlings in a population (<5%, [58]) may have adult-like underwing plumage, the majority of yearlings in some populations may have song repertoires that are indistinguishable from those of local adults [5], [12]. Although each of these traits (song repertoire and underwing plumage) has the potential to provide information about a male's age, assessing them together increases the likelihood that a female will choose optimally and mate with an adult male [2], [36], [64], [65]. Whether females actually use age-related plumage differences in mate choice is not known and this question is a major focus of our current AV studies.

## Supporting Information

Video S1 **A normal speed recording of a male brown-headed cowbird directing a wing-spread song display at a conspecific male.** Video recordings S1–S4 are at lower resolution than the original avi recordings used as playback in the study and were compressed and converted to wmv format for internet presentation. They include the original sound tracks which were replaced by higher quality recordings when preparing the playback stimuli as describe in the Materials and Methods section above. The sound quality is poor because the audio was recorded using the webcam's microphone and in conditions where there was a lot of background noise. The intended recipient of the display in these recordings was located behind the camera.
(WMV)

Video S2 **A normal speed recording of a male brown-headed cowbird directing a wing-spread song display at a conspecific female.**
(WMV)

Video S3 **A slow-motion (half-speed) recording of a male brown-headed cowbird directing a wing-spread song display at a conspecific male.**
(WMV)

Video S4 **A slow motion (half-speed) recording of a male brown-headed cowbird directing a wing-spread song display at a conspecific female.**
(WMV)
